# Yellow perch genetic structure and habitat use among connected habitats in eastern Lake Michigan

**DOI:** 10.1002/ece3.5219

**Published:** 2019-07-23

**Authors:** Gregory M. Chorak, Carl R. Ruetz, Ryan A. Thum, Charlyn G. Partridge, David J. Janetski, Tomas O. Höök, David F. Clapp

**Affiliations:** ^1^ Robert B. Annis Water Resources Institute Grand Valley State University Muskegon Michigan; ^2^ Department of Plant Sciences and Plant Pathology Montana State University Bozeman Montana; ^3^ Department of Biology Indiana University of Pennsylvania Indiana Pennsylvania; ^4^ Department of Forestry and Natural Resources Illinois‐Indiana Sea Grant Purdue University West Lafayette Indiana; ^5^ Charlevoix Fisheries Research Station Michigan Department of Natural Resources Charlevoix Michigan

**Keywords:** drowned river mouth lakes, microsatellites, *Perca flavescens*, population

## Abstract

Maintenance of genetic and phenotypic diversity is widely recognized as an important conservation priority, yet managers often lack basic information about spatial patterns of population structure and its relationship with habitat heterogeneity and species movement within it. To address this knowledge gap, we focused on the economically and ecologically prominent yellow perch (*Perca flavescens*). In the Lake Michigan basin, yellow perch reside in nearshore Lake Michigan, including drowned river mouths (DRMs)—protected, lake‐like habitats that link tributaries to Lake Michigan. The goal of this study was to examine the extent that population structure is associated with Great Lakes connected habitats (i.e., DRMs) in a mobile fish species using yellow perch as a model. Specifically, we tested whether DRMs and eastern Lake Michigan constitute distinct genetic stocks of yellow perch, and if so, whether those stocks migrate between the two connected habitats throughout the year. To do so, we genotyped yellow perch at 14 microsatellite loci collected from 10 DRMs in both deep and littoral habitats during spring, summer, and autumn and two nearshore sites in Lake Michigan (spring and autumn) during 2015–2016 and supplemented our sampling with fish collected in 2013. We found that yellow perch from littoral‐DRM habitats were genetically distinct from fish captured in nearshore Lake Michigan. Our data also suggested that Lake Michigan yellow perch likely use deep‐DRM habitats during autumn. Further, we found genetic structuring among DRMs. These patterns support hypotheses of fishery managers that yellow perch seasonally migrate to and from Lake Michigan, yet, interestingly, these fish do not appear to interbreed with littoral fish despite occupying the same DRM. We recommend that fisheries managers account for this complex population structure and movement when setting fishing regulations and assessing the effects of harvest in Lake Michigan.

## 1 INTRODUCTION

Understanding population genetic structure is crucial for conservation and management of fishes. Many fish species represent genetically and phenotypically diverse stocks (subpopulations with attributes relevant to their management) that were not recognized in many species before molecular population genetics was applied to fisheries management (Begg, Friedland, & Pearce, 1999; Stephenson, 1999). This diversity is important to fisheries management because it can stabilize populations of exploited species to natural and human‐induced disturbances (Schindler et al., 2010; Schindler, Armstrong, & Reed, 2015). For example, historically minor producing stocks of the sockeye salmon (*Oncorhynchus nerka*) fishery in Bristol Bay, Alaska, became the dominant producers of the fishery after climate warming in the past 20 years, highlighting the need to conserve stock diversity in exploited fishes (Hilborn, Quinn, Schindler, & Rogers, 2003). However, most fish stocks are not as easy to identify as salmonids; therefore, population genetics is central for identifying cryptic stocks (distinct stocks of an exploited species residing in sympatry) in the sustainable management of exploited fishes.

Cryptic stock sorting may occur in species whose ranges span a variety of connected habitats that allow distinct stocks to reside in sympatry during different seasons or stages of the life cycle (Brenden et al., 2015; Hilborn et al., 2003; Wilson, Liskauskas, & Wozney, 2016). This is relevant to management, because unexpected harvest of a specific stock can occur when the stock is harvested in a location that is used seasonally and/or during a specific life stage migration. For instance, stocks of walleye (*Sander vitreus*) from Lake St. Clair migrate into Saginaw Bay, Lake Huron. Genetic analysis of harvested walleye indicated that 26% of the total catch taken from Saginaw Bay in 2008 and 2009 was Lake Huron and Lake St. Clair fish that were not accounted for in their respective management units (Brenden et al., 2015). Therefore, understanding cryptic stock sorting is especially important where different stocks can reside in sympatry during seasons where harvesting occurs.

Yellow perch (*Perca flavescens*) is an ecologically valuable and exploited fish species in North America that has long been a focus of fisheries managers (Scott & Crossman, 1973; Becker, 1983). Yellow perch normally spawn in the spring shortly after ice‐out over a 1‐ to 3‐week timeframe, have relatively unspecialized requirements for spawning substrate in slow‐moving or standing waters, and do not construct nests or guard their eggs and young (Becker, 1983). The species once provided a prominent commercial fishery in the Laurentian Great Lakes (avg. 1.1 million kg/year in Lake Michigan during 1889–1970; Becker, 1983). Yet, yellow perch suffered dramatic declines in recruitment in the late 1980s across the Great Lakes and commercial fishing has since slowed dramatically (Marsden & Robillard, 2004). However, there are still substantial numbers of yellow perch harvested across the Great Lakes by recreational anglers each year (200,000–400,000 fish/year in Southern Lake Michigan; Clapp, Elliott, Lenart, & Claramunt, 2012).

Yellow perch has since remained at a much lower abundance in Lake Michigan than historical levels (Clapp & Dettmers, 2004). Yellow perch reside in both Lake Michigan and connecting drowned river mouths (DRMs; Janetski, Ruetz, Bhagat, & Clapp, 2013). DRMs are lake‐like habitats that connect tributaries to Lake Michigan (Janetski & Ruetz, 2015), and receive inputs of water and nutrients from both the tributary and Lake Michigan (Larson et al., 2013; Liu et al., 2018; Wilcox et al., 2002). Recreational harvest of yellow perch in Lake Michigan and DRMs was managed as distinct units with lower limits in Lake Michigan (35 fish/day) than most DRMs (50 fish/day; MDNR, 2016), although a statewide harvest limit of 25 fish/day was enacted during the 2019 fishing season. We know that yellow perch exhibit genetic structure at large, geographic scales corresponding to recolonization of most of the Great Lakes from the Mississippian refugium and eastern lakes from the Atlantic refugium (Sepulveda‐Villet & Stepien, 2012). At finer spatial scales, there also is structuring of yellow perch in Lake Erie and Lake Michigan (Glover, Dettmers, Wahl, & Clapp, 2008; Miller, 2003; Sepulveda‐Villet, Stepien, & Vinebrooke, 2011). However, the question remains whether yellow perch in DRMs are genetically distinct stocks from Lake Michigan, and if so, whether these stocks ever reside in sympatry, such that harvest in one habitat might result in unaccounted take from a different stock.

Previous studies of yellow perch morphology, movement, and genetics provide evidence that DRMs may represent distinct stocks from Lake Michigan and that those stocks may mix at certain times of the year. Fisheries managers hypothesize that larger, lighter‐colored yellow perch (sometimes termed “white bellies”) that are caught by recreational anglers in DRMs during autumn and winter are yellow perch that seasonally migrate from Lake Michigan into DRMs (Schneider, O'Neal, & Clark, 2007). Yellow perch may migrate into DRMs in late autumn and winter given the higher productivity of DRMs relative to nearshore Lake Michigan (Höök, Rutherford, Mason, & Carter, 2007; Janetski & Ruetz, 2015) for feeding and possibly spawning. These anecdotal accounts are corroborated by a recent otolith microchemistry study that found evidence for at least two types of yellow perch: resident DRM (which use DRM wetlands throughout life) and Lake Michigan fish that appear to return to DRM wetlands annually (Schoen, Student, Hoffman, Sierszen, & Uzarski, 2016). Morphological and genetic differences also were found between yellow perch captured in Lake Michigan and DRM wetlands (Parker, Stepien, Sepulveda‐Villet, Ruehl, & Uzarski, 2009); however, the number of fish and DRMs sampled previously for genetic assessment was inadequate to answer whether true stock divisions exist. Therefore, the existence of stock divisions in yellow perch between these connected habitats, and the extent that stocks may move between DRMs and Lake Michigan, remains unclear.

Our goal was to assess the stock structure of the exploited yellow perch in eastern Lake Michigan both spatially and temporally. We aimed to address three questions: (a) Are Lake Michigan yellow perch genetically distinct stocks from DRM yellow perch? (b) If so, do yellow perch from Lake Michigan stocks use DRM habitats during any season where they may be harvested at the higher rate allowed in DRMs? and (c) Are yellow perch stocks distinct between DRM lakes? To evaluate these questions, we sampled fish for microsatellite analysis in deep and littoral habitats of DRMs in spring, summer, and autumn. We also sampled yellow perch in nearshore Lake Michigan in autumn and spring seasons.

## 2 METHODS

### 2.1 Field sites and sample collections

**Figure 1:**
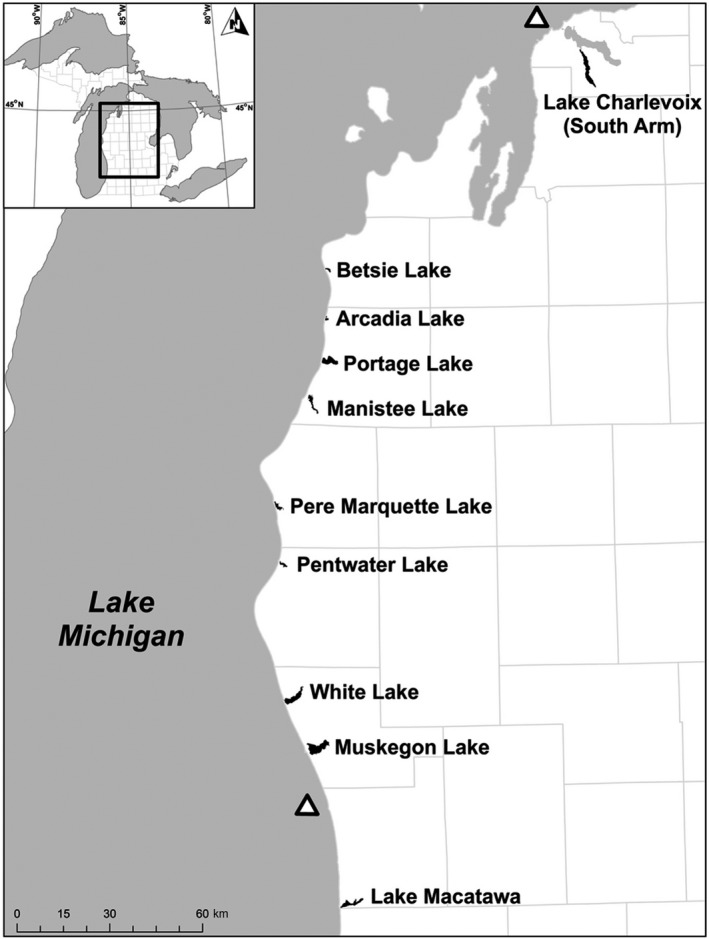
Map of eastern Lake Michigan showing drowned river mouth lakes sampled for yellow perch in deep and littoral habitats between summer 2015 and autumn 2016. Triangles indicate the two nearshore Lake Michigan sampling locations

In an effort to sample possible resident and transient yellow perch in DRM lakes, we collected yellow perch in both deep and littoral habitats of 10 DRMs along the eastern shore of Lake Michigan (Figure 1). We hypothesized that Lake Michigan fish use the more productive deep‐DRM habitats (Höök et al., 2007; Janetski & Ruetz, 2015) during late autumn and winter (prior to the spawning season), when oxygen concentrations and temperatures of the deep‐DRM are most similar to nearshore Lake Michigan (Altenritter, Wieten, Ruetz, & Smith, 2013; Biddanda et al., 2018; Weinke & Biddanda, 2018). Yellow perch from deep‐DRM habitats were captured using 5.08‐ and 7.62‐cm stretch‐mesh gill nets placed on the bottom in the deepest part (range = 8.4–20.5 m) of each DRM where dissolved oxygen concentration was >2 mg/ml. Littoral‐DRM habitats were sampled using boat electrofishing. We divided the shoreline of each DRM into 200‐m numbered transects, and then, three transects were selected randomly and electrofished for 20 min. If the target number of yellow perch (40 individuals) was not achieved at the randomly selected transects, additional transects were chosen based on habitat (e.g., presence of submerged aquatic vegetation) to reach the target number of fish.

To account for temporal patterns of habitat use by yellow perch, we sampled fish from deep and littoral habitats of DRM lakes during spring, summer, and autumn 2015–2016, although not all DRMs or habitats were sampled in every season (see Table 1). We defined summer as when the DRM lake was thermally stratified, autumn as after turnover (i.e., loss of thermal stratification) and before ice cover, and spring as after ice‐out and before thermal stratification.

**Table 1:** Number of yellow perch collected by habitat (nearshore Lake Michigan, littoral‐drowned river mouth [DRM], or deep‐DRM), season, and year at two sites in Lake Michigan (LM) and 10 DRMs

Site	Season	Year	No. of Yellow Perch		
			Nearshore	Littoral‐DRM	Deep‐DRM
Northern LM	Spring	2016	19		
	Autumn	2013	40		
Charlevoix	Summer	2015		39	0
	Autumn	2015		0	2
Betsie	Spring	2016		27	0
	Summer	2015		28	0
Arcadia	Spring	2016		40	0
	Summer	2015		40	0
Portage	Summer	2015		40	0
	Autumn	2015		5	0
Manistee	Spring	2016		40	0
	Summer	2015		40	0
Pere Marquette	Spring	2016		40	3
	Summer	2015		40	10
Pentwater	Spring	2016		40	0
	Summer	2015		40	1
	Autumn	2015, 2016		0	47
White	Spring	2016		40	10
	Summer	2015		40	0
	Autumn	2016		0	67
Muskegon	Spring	2016		25	0
	Summer	2015, 2016		40	4
	Autumn	2013, 2015, 2016		20	68
Macatawa	Summer	2015, 2016		58	–
	Autumn	2016		–	9
Southern LM	Summer	2016	40		
	Autumn	2013	20		

Notes

In total, 1,022 yellow perch were captured and genotyped, but after quality control, only 975 individuals were included in analyses (see Table S2). “–” indicates that the habitat was not sampled, whereas “0” indicates that habitat was sampled but no fish were captured.

Yellow perch were captured in nearshore Lake Michigan by the Michigan Department of Natural Resources (MDNR) during late summer and spring seasons (2016) using gill nets and trawling as part of their biannual survey of yellow perch (Fitzgerald, Clapp, & Belonger, 2004; Fetzer et al., 2017). Two sites were sampled in nearshore Lake Michigan. One site was adjacent to the furthest north DRM, Charlevoix, and the other site was located between the two most southern DRMs, Macatawa and Muskegon (Figure 1). We supplemented our sampling with yellow perch fin clips collected from northern and southern Lake Michigan by MDNR and from the deep‐DRM habitat in Muskegon Lake during 2013 as part of another project (see Harris, Ruetz, Wieten, Altenritter, & Smith, 2017). These fin clips were genotyped with the fish collected for this study.

## 3 MOLECULAR METHODS

We clipped tissue from the anal fin of each yellow perch. Clips were either stored in ethanol or dried in a scale envelope. We then extracted whole DNA from ~4 mm^2^ of fin tissue using a modified method from Walsh, Metzger, and Higuchi (1991). Approximately 30% volume of Chelex 100 (Sigma‐Aldrich), 0.112 µg proteinase K, and ultrapure water were combined for 150 µl total extraction volume. We incubated fin clips in extraction buffer at 76°C for 1 hr and 99°C for 10 min.

We initially amplified 16 microsatellite markers in each individual. Microsatellite markers used here were previously developed for yellow perch (YP: Li, Wang, Givens, Czesny, & Brown, 2006; Pfla: Leclerc, Wirth, & Bernatchez, 2000; and Mpf: Grzybowski et al., 2010) and walleye (Svi: Borer, Miller, & Kapuscinski, 1999). We performed polymerase chain reaction (PCR) to amplify each locus in 25 µl total reaction volume consisting of 4X KCl Buffer (Thermo Sci.), 2 mM MgCl_2_, 0.2 mM of each dNTP (New England Biolabs), 1 µM each primer (dye‐labeled forward and reverse), 1.25U *Taq* DNA Polymerase (Thermo Sci.), and ~ 100 ng template DNA. All amplifications started at 95°C for 3 min followed by 30 cycles of 95°C for 30 s, an annealing step for 1 min (temperatures varied, see Table S1), and 72°C for 30 s. A final extension for 10 min at 72°C finished the amplification. The exceptions to this method were a touchdown PCR on Svi‐6 and an extra 5 cycles on Pfla‐L6 (see Table S1). We visualized microsatellite markers on a 3130xl genetic analyzer using HiDi chemistry (Applied Biosystems).

### 3.1 Data analysis

We scored markers blindly to their collection location in GeneMapper v5 (Applied Biosystems). In total, we collected DNA from 1,022 yellow perch. We performed quality control using the STRATAG package (Archer, Adams, & Schneiders, 2017) in R, where we removed samples missing ≥80% of loci and any site–habitat grouping with <15 individuals to avoid allele frequency mischaracterization due to sample size. We then grouped populations by site and habitat (deep‐DRM, littoral‐DRM, or nearshore Lake Michigan) and tested conformity of loci to Hardy–Weinberg equilibrium (HWE) and linkage disequilibrium in Genepop v4.2 (Raymond & Rousset, 1995) using 100 batches of 1,000 Markov Chain Monte Carlo (MCMC) iterations.

After filtering the data to remove site–habitat groupings with small sample size and individuals with missing loci, our dataset contained 975 yellow perch for analyses: 187 from deep‐DRM habitats, 681 from littoral‐DRM habitats, and 107 from nearshore Lake Michigan. We only included deep‐DRM habitat fish collected in autumn for analyses (Table 1). This was because we captured few yellow perch in deep‐DRM habitats during spring and summer seasons; therefore, sample sizes were not sufficient to make meaningful comparisons with the other site–habitat groupings. Sample sizes of DRMs, both deep and littoral habitats, ranged from 39 to 84 individuals (see Table S2). In Lake Michigan, the sample size was 60 fish at the southern site and 47 fish at the northern site.

Two loci (Pfla‐L3 and Pfla‐L4) had intense stutter in their chromatograms, which likely caused unreliable genotype scoring. These loci were excluded from analyses because they were out of HWE in more than 60% of the site–habitat groupings. Yellow perch collected in Lake Michigan and Muskegon deep in 2013 were not different (based on *F*_ST_ and clustering analyses of microsatellites) from our samples (years 2013 vs. 2016), so fish were pooled across years within sites in the analyses reported below. The same was true of fish collected at the same site between spring and summer seasons; samples were combined from those seasons into “littoral‐DRM” group. No loci showed evidence of linkage disequilibrium within a site–habitat group. However, six loci (YP60, YP78, YP96, Pfla‐L6, Svi4, and Mpf4) showed linkage disequilibrium when all populations were combined, likely due to population structure in the dataset.

We assessed individual‐level genetic clustering using the Bayesian clustering program STRUCTURE v2.3.2 (Pritchard, Stephens, & Donnelly, 2000). Yellow perch were clustered using the admixture model, λ = 1, and a burn‐in period of 100,000 and a run time of 200,000 MCMC reps, 10 iterations at each value of K (1–17). We ran STRUCTURE both with and without priors. We used sampling location (site–habitat grouping) as a priori population indicators. We found the most likely values of K using the ΔK method from Evanno, Regnaut, and Goudet (2005) calculated in STRUCTURE HARVESTER v0.6.93 (Earl & vonHoldt, 2012), which are reported in Figure S1. We found consensus clusters across iterations of STRUCTURE by permuting and matching clusters using the large K greedy algorithm with a random input and 1,000 repeats in CLUMPP v1.1.2 (Jakobsson & Rosenberg, 2007), and we used *distruct* v1.1 (Rosenberg, 2004) to draw the final STRUCTURE plots.

We performed further clustering of individuals combining sites by habitat type (deep‐DRM, littoral‐DRM, nearshore Lake Michigan) and using discriminant analysis of principal components (DAPC) in the *adegenet* v2.0.1 (Jombart, 2008) package for R. DAPC uses a multivariate approach that may reveal complex structuring better than Bayesian clustering (Jombart, Devillard, & Francois, 2010). We used DAPC to determine which habitat (littoral‐DRM or nearshore Lake Michigan) the yellow perch captured in the deep‐DRM habitats clustered with, to evaluate our hypothesis that yellow perch in deep‐DRM habitats during autumn may be transient Lake Michigan fish.

We calculated pairwise *F*_ST _(Weir & Cockerham, 1984) between all site–habitat groupings in STRATAG (Archer et al., 2017) to test whether sampling locations were genetically distinct. We applied a Holm–Bonferroni sequential correction (Holm, 1979) to pairwise *F*_ST _calculations to correct for multiple comparisons, which we recognize is conservative but did not affect our conclusions given the magnitude of the differences. Finally, we tested for isolation by distance (IBD) using the natural logarithm distances between DRMs and the linearized pairwise *F*_ST_ (Slatkin, 1993) comparisons of yellow perch collected in littoral‐DRM habitats using a Mantel test with 999 replicates in the R package *adegenet* v2.0.1 (Jombart, 2008). We used PGDSpider v2.1.1.0 software (Lischer & Excoffier, 2012) to convert our data between different dataset formats.

## 4 RESULTS

The overall *F*_ST_ was 0.024. Marker diversity was higher among DRM lakes (9.79–13.43 average alleles/locus) compared to Lake Michigan sites (9.64–9.93 avg. alleles/locus; Table S2). Private alleles ranged between zero and seven within a population, and heterozygosity within populations was fairly even (0.53–0.65; Table S2).

**Figure 2:**
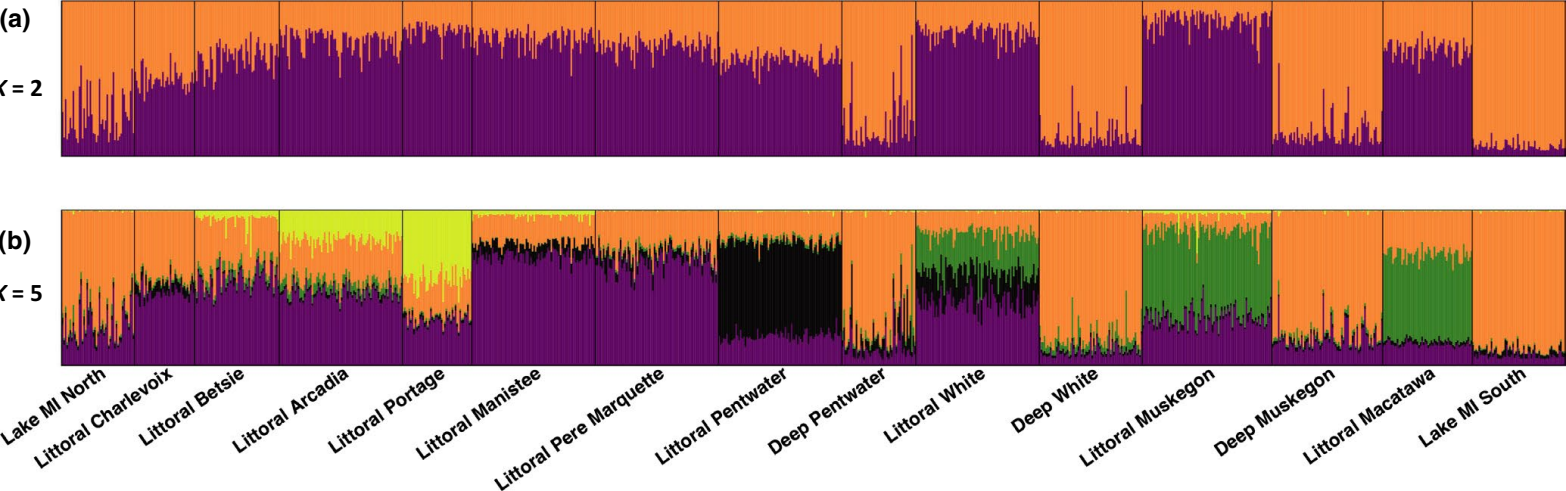
Distruct plot of STRUCTURE cluster assignments for each yellow perch (*n* = 975). Sampling locations listed were used as a priori population indicators. Each vertical bar represents one fish, and color indicates the probability of an individual belonging to one of two (a) or five (b) genetic clusters. Dark vertical bars separate lake/habitats and lakes are ordered north to south from left to right

**Figure 3:**
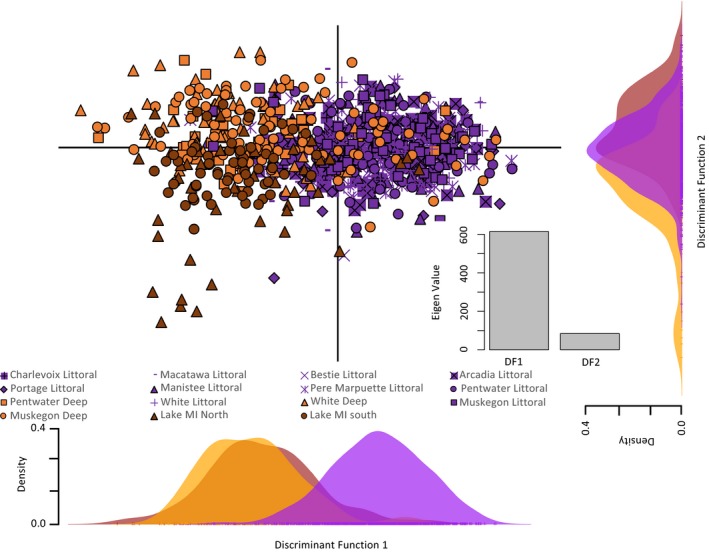
First two axes of discriminant analysis of principal components (DAPC) of genotypes for all individuals (*n* = 975). Each dot represents an individual, and symbols represent sampling location. Individuals are also color‐coded and grouped by habitat type. Lower right of plot shows eigenvalues of two axes relative to one another. Each single‐axis distribution is plotted outside corresponding ordination axes

We found littoral‐DRM and Lake Michigan yellow perch to be distinct from one another. In our STRUCTURE analysis at *K* = 2, Lake Michigan yellow perch and littoral‐DRM yellow perch clearly exhibited different group memberships (Figure 2a), and this general pattern held when we did not use location as prior and as K increased (Figure S2; Figure 2b). Similar to STRUCTURE, the results of DAPC showed distinct clusters between individuals sampled in littoral‐DRMs and Lake Michigan habitats (Figure 3) and no alleles weighed disproportionately on either axis of the ordination (Table S3). Further, yellow perch from all littoral‐DRM habitats were significantly different from both Lake Michigan sites in pairwise F_ST_ comparisons (Table 2).

**Table 2:** Pairwise F_ST_ among littoral‐DRM habitats, deep‐DRM habitats, and nearshore Lake Michigan sites

	N. Lake MI	Littoral Charlevoix	Littoral Betsie	Littoral Arcadia	Littoral Portage	Littoral Manistee	Littoral Pere Marquette	Littoral Pentwater	Deep Pentwater	Littoral White	Deep White	Littoral Muskegon	Deep Muskegon	Littoral Macatawa
Littoral Charlevoix	0.008*	–												
Littoral Betsie	0.019*	0.015*	–											
Littoral Arcadia	0.022*	0.022*	0.008*	–										
Littoral Portage	0.025*	0.027*	0.014*	0.009*	–									
Littoral Manistee	0.032*	0.028*	0.016*	0.009*	0.020*	–								
Littoral Pere Marquette	0.018*	0.017*	0.014*	0.011*	0.024*	0.017*	–							
Littoral Pentwater	0.030*	0.030*	0.022*	0.023*	0.031*	0.020*	0.021*	–						
Deep Pentwater	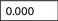	0.022*	0.024*	0.024*	0.028*	0.034*	0.025*	0.027*	–					
Littoral White	0.027*	0.025*	0.016*	0.011*	0.021*	0.015*	0.010*	0.011*	0.031*	–				
Deep White	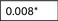	0.028*	0.028*	0.027*	0.031*	0.031*	0.028*	0.024*	0.001	0.032*	–			
Littoral Muskegon	0.030*	0.027*	0.011*	0.007*	0.018*	0.010*	0.009*	0.014*	0.032*	0.006*	0.030*	–		
Deep Muskegon	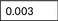	0.025*	0.027*	0.027*	0.032*	0.029*	0.026*	0.028*	0.000	0.032*	−0.001	0.031*	–	
Littoral Macatawa	0.022*	0.020*	0.018*	0.016*	0.027*	0.019*	0.007*	0.019*	0.029*	0.011*	0.029*	0.009*	0.030*	–
S. Lake MI	0.008*	0.035*	0.037*	0.043*	0.043*	0.053*	0.043*	0.041*	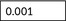	0.049*	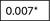	0.051*	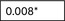	0.046*

Notes

All values represent pairwise *F*_ST_ scores, and values with * are statistically significant (*p* < 0.05) after Holm–Bonferroni sequential correction. Outlined values show comparisons between Lake Michigan and deep‐DRM. Dark highlighted values represent comparisons between deep and littoral habitats in the same DRM, and light highlighted values represent comparisons between deep habitats among DRMs.

Deep‐DRM yellow perch were genetically distinct from littoral‐DRM fish but were not distinct from Lake Michigan fish. At *K* = 2 and 5, most yellow perch from deep‐DRM habitats and Lake Michigan sites belonged to the same genetic cluster (majority “orange” cluster; Figure 2). DAPC clustering of deep‐DRM fish supported this clustering found in STRUCTURE. The most informative axis, Discriminant Function 1 (based on eigenvalues for both axes of DAPC), suggests that yellow perch from deep‐DRM habitats and Lake Michigan may belong to the same genetic cluster. However, Discriminant Function 2 separates deep‐DRM and Lake Michigan yellow perch, but with much less magnitude based on the eigenvalue of that axis (Figure 3). With only three groups and plotting two axes, the second discriminant function maximizes any separation between the deep‐DRM and Lake Michigan groups, so particular attention should be paid to the eigenvalues of each function of the DAPC for proper inference (Figure 3). Pairwise *F*_ST_ comparisons were generally congruent with findings from DAPC and STRUCTURE. Average *F*_ST_ between littoral‐DRM habitats and nearshore Lake Michigan was much higher (mean and median ≈ 0.034) than between deep‐DRM habitats and nearshore Lake Michigan (mean and median ≈ 0.005), supporting the grouping of Lake Michigan and deep‐DRM fish together but distinct from littoral‐DRM yellow perch in the clustering analyses. However, yellow perch from the deep‐water habitats in DRMs were not always significantly different from Lake Michigan sites based on *F*_ST_. The deep‐water fish from Muskegon Lake were not significantly different from northern Lake Michigan, and yellow perch from the deep‐water habitat of Pentwater Lake were not significantly different from either northern or southern Lake Michigan sites in pairwise *F*_ST_ comparisons (Table 2). The deep habitat of White Lake was significantly different from both Lake Michigan sites, and the deep habitat of Muskegon Lake was significantly different from southern Lake Michigan, although values of *F*_ST _were small (range = 0.007–0.008) for each comparison. Additionally, the divergence between the northern and southern Lake Michigan sites was small but significant (*F*_ST_ = 0.008, Table 2).

**Figure 4:**
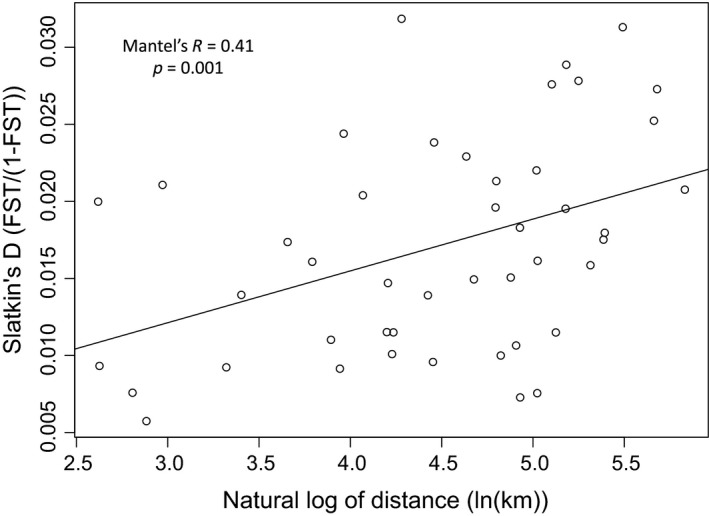
Association between linearized genetic difference (*F*_ST_/1 − *F*_ST_; Slatkin, 1993) and the natural log of geographic distance (ln(km)) among all pairwise comparisons of drowned river mouth yellow perch. Only yellow perch from littoral‐DRM habitats were used in the Mantel test (*n* = 681). The line represents the best fit of the linear model

Finally, littoral‐DRM yellow perch were distinct between DRMs. Although pairwise *F*_ST _values were low (mean ≈ 0.017, median ≈ 0.016), all yellow perch from littoral‐DRM habitats were significantly different from one another (Table 2). Further, there was a significant pattern of isolation by distance between DRMs (Mantel's *R* = 0.41, *p* = 0.001; Figure 4). When STRUCTURE was run for five genetic groups (*K* = 5), littoral‐DRM lakes clustered distinctly from one another and lakes geographically close to each other shared clusters (Figures 1, 2b), supporting the results of pairwise *F*_ST_ and the Mantel test. However, the clusters were not clearly defined, possibly due to the continuous isolation‐by‐distance pattern exhibited between DRMs.

## 5 DISCUSSION

Our results suggest that yellow perch in littoral‐DRMs form distinct stocks from eastern Lake Michigan. Although divergences between littoral‐DRM populations and Lake Michigan fish were small (*F*_ST_: Charlevoix = 0.008; others = 0.018–0.053), all of the differences were significant. Furthermore, both STRUCTURE and DAPC analyses consistently grouped littoral‐DRM yellow perch distinctly from Lake Michigan yellow perch (Figures 2, 3). Our finding that littoral‐DRM and Lake Michigan yellow perch are distinct genetic stocks is supported by previous otolith and morphology studies that suggested these may be distinct stocks (Parker et al., 2009; Schoen et al., 2016).

Although littoral‐DRM and Lake Michigan yellow perch are genetically distinct stocks, we found evidence that Lake Michigan yellow perch use deep‐DRM habitats. Both STRUCTURE and DAPC grouped yellow perch collected in deep‐DRM habitats during autumn with Lake Michigan fish (see Figures 2, 3). Our findings support the hypothesis that some Lake Michigan yellow perch migrate into DRMs in autumn and winter (prior to the spring spawning season), which was further supported by a recent study of otolith microchemistry that found evidence that yellow perch can primarily reside in Lake Michigan and make annual migrations into DRM wetlands (Schoen et al., 2016).

The seasonal sympatry of genetically distinct stocks of yellow perch in DRMs has important management implications. Catch limits for Lake Michigan yellow perch were lower than for DRMs prior to 2019 (35 vs. 50 fish/day, respectively; MDNR, 2016). Although yellow perch harvest limits were changed to be 25 fish/day in Michigan during the 2019 fish season, the population impacts of harvesting Lake Michigan fish in DRMs during autumn and winter are uncertain but warrant further investigation. Recreational harvest of yellow perch in autumn and winter is extensive in many DRMs. For instance, anglers harvested over 50,000 yellow perch from Muskegon Lake in one winter season (in 2003; Hanchin, O'Neal, Clark, & Lockwook, 2007). Therefore, we recommend fishery managers account for the proportion of Lake Michigan yellow perch harvested by anglers in DRMs during autumn and winter to determine whether these harvests significantly impact population dynamics of Lake Michigan stocks.

The reasons for the annual migration of Lake Michigan yellow perch into DRMs are unclear. One explanation may be that Lake Michigan yellow perch utilize DRMs for foraging habitats because prey densities are higher than in Lake Michigan (Höök et al., 2007). Both managers and researchers have suggested that yellow perch from Lake Michigan may migrate into DRMs to spawn (Perrone, Schneeberger, & Jude, 1983; Schneider et al., 2007; Schoen et al., 2016). If so, then the genetic divergence of yellow perch captured in littoral‐DRM habitats versus Lake Michigan suggests that Lake Michigan yellow perch either predominantly spawn in different locations or at different times than littoral‐DRM fish.

Regardless of the reasons for migration of Lake Michigan fish into DRMs, yellow perch appear to begin entering deep‐DRM habitats in autumn and return to Lake Michigan by the spring since we did not catch Lake Michigan fish in DRMs during our spring sampling (see Table 1). This timing of the migration may reflect when environmental conditions in deep‐DRM habitats match the conditions of Lake Michigan (Altenritter et al., 2013; Weinke & Biddanda, 2018; Biddanda et al., 2018). For instance, dissolved oxygen concentrations often become lower in the deepest parts of DRMs during summer when the water is thermally stratified (Figure S3; see Altenritter et al., 2013; Weinke & Biddanda, 2018; Biddanda et al., 2018). Future studies implementing telemetry (Hayden et al., 2014) could elucidate the timing and duration of DRM use by Lake Michigan yellow perch, which may facilitate accounting for the harvest of Lake Michigan fish in DRMs. Moreover, studies of otolith isotopes (Dufour, Patterson, Höök, & Rutherford, 2005; Dufour, Höök, Patterson, & Rutherford, 2008) or trace elements (Schoen et al., 2016) could provide insights on movement patterns between the two habitats.

Our results also suggest that there is a portfolio of yellow perch genetic diversity in the DRMs of eastern Lake Michigan. Previously, yellow perch stocks were found to be distinct between Green Bay and the southern basin of Lake Michigan (Miller, 2003; Glover et al., 2008). However, we found littoral yellow perch populations from all DRMs exhibited significant differences based on pairwise *F*_ST_ (Table 2), suggesting that yellow perch population structure is more complex than originally reported in Lake Michigan. Furthermore, genetic divergence between DRMs exhibited a significant pattern of isolation by distance (Figure 4), which was similar to the pattern found for invasive round goby (*Neogobius melanostomus*) among Lake Michigan pierheads (LaRue, Ruetz, Stacey, & Thum, 2011). Therefore, we hypothesize that DRMs are loosely distinct populations connected by gene flow. The importance of this underlying population structure to fisheries management is unclear; however, managers should think about yellow perch genetic diversity within and among DRMs when setting harvest regulations.

In conclusion, understanding population structure of species residing in connected habitats and in sympatry at certain times of the year and/or life cycle has proved critical for maintaining sustainable fisheries (Hilborn et al., 2003; Brenden et al., 2015; Wilson et al., 2016). Here, we showed that yellow perch from Lake Michigan and DRMs represent distinct stocks and that Lake Michigan yellow perch can reside in DRMs during autumn, where they may be subjected to greater harvest than in Lake Michigan. Maintaining yellow perch stock diversity may therefore depend on where they reside both spatially and temporally, and future studies should aim to support management strategies that will preserve yellow perch stocks. Until then, we recommend that results from our study be considered when managing yellow perch and setting harvest limits in Lake Michigan, especially with respect to DRMs.

## CONFLICT OF INTEREST

None declared.

## AUTHOR CONTRIBUTIONS

G.M.C. led this project and carried out all aspects of it. C.R.R., R.A.T., D.J.J., T.O.H., and D.F.C. conceived the project, acquired the funding, and contributed significantly along the course of the project and with writing. D.F.C. collected samples from Lake MI. C.G.P. contributed significantly in the laboratory and with writing.

## DATA AVAILABILITY STATEMENT

Microsatellite genotypes from this study are available at Dryad https://doi.org/10.5061/dryad.2444ck7.
