# Supplementary material for: Yellow perch genetic structure and habitat use among connected habitats in eastern Lake Michigan
Source: Ecol Evol. 2019 Jul 23;9(16):8922–32. doi: 10.1002/ece3.5219 (PMC6706189; doi:10.1002/ece3.5219)
Supplement: Supplementary file 1 [file ECE3-9-8922-s001.pdf]

Supporting Tables

Supplemental Table S1. Summary of PCR parameters and locus statistics for 14 microsatellite loci.

**Loci**	Num. Genotyped	Num. Alleles	Obsvd. Heterozygosity	Expt. Heterozygosity	Annealing Temp. (ºC)	Cycles	Source	Dye-Label
**Mpf.4**	956	29	0.77	0.78	54	35	Gryzbowski et al. 2010	FAM
**Mpf.5**	973	14	0.55	0.56	54	35	Gryzbowski et al. 2010	FAM
**Mpf.6**	958	10	0.54	0.55	54	35	Gryzbowski et al. 2010	VIC
**Mpf.7**	968	30	0.86	0.88	54	35	Gryzbowski et al. 2010	VIC
**Pfla.L2**	944	15	0.53	0.56	51	35	Leclerc et al. 2000	PET
**Pfla.L5**	963	14	0.48	0.49	51	35	Leclerc et al. 2000	VIC
**Pfla.L6**	920	18	0.46	0.49	47	40	Leclerc et al. 2000	FAM
**Svi.33**	901	46	0.91	0.96	61	35	Borer et al. 1999	NED
**Svi.4**	958	30	0.94	0.88	61	35	Borer et al. 1999	FAM
**Svi.6**	916	41	0.79	0.91	TD (65,55)	10@65, 30@55	Borer et al. 1999	VIC
**YP41**	956	8	0.54	0.54	54	35	Li et al. 2006	FAM
**YP60**	962	9	0.31	0.31	51	35	Li et al. 2006	FAM
**YP78**	956	14	0.52	0.52	54	35	Li et al. 2006	NED
**YP96**	963	8	0.19	0.20	51	35	Li et al. 2006	VIC

Supplemental Table S2. Summary of population differentiation statistics by sampling location.

Strata	*n*	Avg. Samples Missing Data	Avg. Alleles/Locus	Num. Private Alleles	Heterozygosity
Arcadia	80	0.3571	13.43	4	0.608
Betsie	55	0.6429	11.64	1	0.621
Charlevoix	39	0.0714	9.79	2	0.599
LK MI North	47	7.6429	9.64	1	0.528
LK MI South	60	0.5	9.93	0	0.57
Macatawa	58	1.6429	11.64	3	0.596
Manistee	80	0.2857	12.21	3	0.624
Muskegon	84	3	14.29	7	0.65
Muskegon Deep	72	3.0714	11.64	2	0.558
Pentwater	80	0.9286	11.43	3	0.618
Pentwater Deep	48	1.6429	10.43	4	0.55
Pere Marquette	80	1.5714	12	3	0.578
Portage	45	1	11.36	0	0.63
White	80	0.3571	13.43	5	0.636
White Deep	67	2.7143	11.86	2	0.567

Supplemental Table S3. Loadings on each axis of DAPC plot (Fig. 3) by each allele at each locus.

Locus.Allele	LD1	LD2
YP41.184	0.0006	0.0043
YP41.180	0.0025	0.0074
YP41.172	0.0000	0.0001
YP41.188	0.0002	0.0122
YP41.176	0.0043	0.0019
YP41.193	0.0000	0.0006
YP41.-9	0.0021	0.0727
YP41.168	0.0000	0.0000
YP41.197	0.0001	0.0000
YP78.202	0.0002	0.0017
YP78.217	0.0005	0.0000
YP78.214	0.0000	0.0031
YP78.211	0.0005	0.0005
YP78.220	0.0154	0.0004
YP78.205	0.0007	0.0002
YP78.208	0.0119	0.0070
YP78.196	0.0084	0.0138
YP78.190	0.0007	0.0009
YP78.193	0.0002	0.0004
YP78.199	0.0000	0.0029
YP78.223	0.0001	0.0004
YP78.-9	0.0169	0.0003
YP78.187	0.0018	0.0000
YP78.181	0.0000	0.0000
Mpf_5.158	0.0006	0.0004
Mpf_5.147	0.0001	0.0033
Mpf_5.156	0.0032	0.0010
Mpf_5.160	0.0000	0.0067
Mpf_5.164	0.0000	0.0001
Mpf_5.152	0.0084	0.0110
Mpf_5.162	0.0007	0.0004
Mpf_5.145	0.0001	0.0000
Mpf_5.150	0.0006	0.0002
Mpf_5.143	0.0008	0.0002

Mpf_5.154	0.0091	0.0001
Mpf_5.170	0.0001	0.0000
Mpf_5.166	0.0000	0.0000
Mpf_5.-9	0.0000	0.0007
Mpf_5.140	0.0001	0.0000
Mpf_7.170	0.0267	0.0006
Mpf_7.186	0.0339	0.0005
Mpf_7.176	0.0198	0.0096
Mpf_7.172	0.0328	0.0028
Mpf_7.182	0.0027	0.0046
Mpf_7.184	0.0185	0.0013
Mpf_7.206	0.0000	0.0000
Mpf_7.178	0.0066	0.0390
Mpf_7.188	0.0001	0.0179
Mpf_7.168	0.0091	0.0000
Mpf_7.192	0.0005	0.0000
Mpf_7.180	0.0073	0.0059
Mpf_7.196	0.0000	0.0000
Mpf_7.190	0.0000	0.0000
Mpf_7.160	0.0001	0.0007
Mpf_7.174	0.0004	0.0285
Mpf_7.194	0.0000	0.0001
Mpf_7.147	0.0145	0.0000
Mpf_7.142	0.0000	0.0000
Mpf_7.166	0.0002	0.0002
Mpf_7.156	0.0000	0.0000
Mpf_7.198	0.0000	0.0001
Mpf_7.200	0.0000	0.0000
Mpf_7.140	0.0001	0.0000
Mpf_7.162	0.0002	0.0000
Mpf_7.158	0.0000	0.0000
Mpf_7.164	0.0000	0.0000
Mpf_7.202	0.0000	0.0000
Mpf_7.150	0.0000	0.0000
Mpf_7.-9	0.0003	0.0004
Mpf_7.152	0.0000	0.0002
YP60.197	0.0015	0.0006
YP60.201	0.0022	0.0010

YP60.189	0.0014	0.0050
YP60.204	0.0017	0.0001
YP60.193	0.0001	0.0090
YP60.186	0.0000	0.0001
YP60.208	0.0002	0.0003
YP60.-9	0.0000	0.0009
YP60.216	0.0003	0.0000
YP60.183	0.0000	0.0000
YP96.133	0.0084	0.0039
YP96.136	0.0000	0.0201
YP96.130	0.0058	0.0050
YP96.143	0.0001	0.0001
YP96.146	0.0000	0.0004
YP96.139	0.0010	0.0096
YP96.149	0.0000	0.0022
YP96.-9	0.0001	0.0009
YP96.152	0.0000	0.0001
Pfla_L2.210	0.0048	0.0003
Pfla_L2.213	0.0080	0.0080
Pfla_L2.215	0.0000	0.0018
Pfla_L2.212	0.0175	0.0127
Pfla_L2.202	0.0009	0.0008
Pfla_L2.208	0.0000	0.0006
Pfla_L2.204	0.0085	0.0125
Pfla_L2.219	0.0026	0.0001
Pfla_L2.217	0.0000	0.0005
Pfla_L2.-9	0.0026	0.0184
Pfla_L2.223	0.0000	0.0000
Pfla_L2.225	0.0001	0.0000
Pfla_L2.206	0.0000	0.0001
Pfla_L2.245	0.0000	0.0000
Pfla_L2.221	0.0000	0.0000
Pfla_L2.230	0.0000	0.0000
Pfla_L5.152	0.0044	0.0028
Pfla_L5.154	0.0150	0.0014
Pfla_L5.171	0.0000	0.0000
Pfla_L5.156	0.0070	0.0013
Pfla_L5.148	0.0141	0.0000

Pfla_L5.132	0.0038	0.0000
Pfla_L5.-9	0.0022	0.0029
Pfla_L5.159	0.0001	0.0000
Pfla_L5.150	0.0018	0.0001
Pfla_L5.140	0.0000	0.0001
Pfla_L5.145	0.0000	0.0000
Pfla_L5.163	0.0000	0.0000
Pfla_L5.136	0.0000	0.0000
Pfla_L5.161	0.0000	0.0000
Pfla_L5.168	0.0000	0.0001
Pfla_L6.171	0.0043	0.0032
Pfla_L6.173	0.0129	0.0001
Pfla_L6.165	0.0006	0.0279
Pfla_L6.169	0.0002	0.0001
Pfla_L6.175	0.0000	0.0001
Pfla_L6.163	0.0016	0.0052
Pfla_L6.158	0.0001	0.0005
Pfla_L6.152	0.0001	0.0004
Pfla_L6.156	0.0056	0.0024
Pfla_L6.-9	0.0463	0.0054
Pfla_L6.154	0.0026	0.0006
Pfla_L6.167	0.0001	0.0002
Pfla_L6.160	0.0000	0.0004
Pfla_L6.177	0.0000	0.0007
Pfla_L6.140	0.0001	0.0000
Pfla_L6.181	0.0000	0.0002
Pfla_L6.179	0.0000	0.0000
Pfla_L6.132	0.0000	0.0000
Pfla_L6.147	0.0000	0.0001
Svi_4.152	0.0006	0.0034
Svi_4.159	0.0124	0.0005
Svi_4.156	0.0048	0.0012
Svi_4.165	0.0009	0.0043
Svi_4.154	0.0145	0.0003
Svi_4.134	0.0000	0.0005
Svi_4.161	0.0080	0.0180
Svi_4.171	0.0010	0.0029
Svi_4.163	0.0001	0.0000

Svi_4.138	0.0000	0.0000
Svi_4.167	0.0001	0.0426
Svi_4.174	0.0000	0.0015
Svi_4.169	0.0003	0.0110
Svi_4.180	0.0000	0.0001
Svi_4.111	0.0080	0.0060
Svi_4.-9	0.0038	0.0525
Svi_4.145	0.0009	0.0188
Svi_4.148	0.0005	0.0002
Svi_4.176	0.0453	0.0490
Svi_4.115	0.0166	0.0009
Svi_4.140	0.0001	0.0000
Svi_4.184	0.0000	0.0000
Svi_4.150	0.0066	0.0128
Svi_4.143	0.0004	0.0002
Svi_4.136	0.0002	0.0000
Svi_4.178	0.0000	0.0001
Svi_4.182	0.0000	0.0000
Svi_4.130	0.0000	0.0000
Svi_4.121	0.0000	0.0001
Svi_4.113	0.0001	0.0000
Svi_4.132	0.0000	0.0000
Svi_33.144	0.0016	0.0027
Svi_33.148	0.0003	0.0044
Svi_33.125	0.0000	0.0001
Svi_33.129	0.0031	0.0177
Svi_33.137	0.0064	0.0010
Svi_33.139	0.0007	0.0153
Svi_33.150	0.0004	0.0451
Svi_33.172	0.0013	0.0001
Svi_33.112	0.0029	0.0011
Svi_33.154	0.0054	0.0057
Svi_33.133	0.0000	0.0029
Svi_33.152	0.0013	0.0000
Svi_33.122	0.0009	0.0089
Svi_33.142	0.0000	0.0003
Svi_33.160	0.0017	0.0049
Svi_33.120	0.0014	0.0022

Svi_33.146	0.0009	0.0005
Svi_33.135	0.0025	0.0002
Svi_33.158	0.0002	0.0033
Svi_33.131	0.0022	0.0001
Svi_33.184	0.0000	0.0000
Svi_33.110	0.0020	0.0044
Svi_33.168	0.0009	0.0050
Svi_33.162	0.0050	0.0022
Svi_33.166	0.0020	0.0029
Svi_33.174	0.0019	0.0000
Svi_33.127	0.0001	0.0074
Svi_33.170	0.0009	0.0000
Svi_33.176	0.0000	0.0000
Svi_33.164	0.0002	0.0001
Svi_33.116	0.0038	0.0000
Svi_33.-9	0.0002	0.0023
Svi_33.186	0.0000	0.0000
Svi_33.100	0.0001	0.0000
Svi_33.156	0.0004	0.0026
Svi_33.104	0.0000	0.0001
Svi_33.178	0.0000	0.0000
Svi_33.188	0.0000	0.0000
Svi_33.124	0.0001	0.0067
Svi_33.194	0.0000	0.0000
Svi_33.106	0.0001	0.0000
Svi_33.182	0.0000	0.0000
Svi_33.114	0.0003	0.0000
Svi_33.192	0.0000	0.0000
Svi_33.180	0.0000	0.0000
Svi_33.196	0.0000	0.0000
Svi_33.118	0.0000	0.0001
Svi_6.155	0.0313	0.0008
Svi_6.189	0.0014	0.0014
Svi_6.186	0.0002	0.0000
Svi_6.168	0.0101	0.0009
Svi_6.200	0.0059	0.0001
Svi_6.176	0.0008	0.0002
Svi_6.204	0.0001	0.0001

Svi_6.172	0.0147	0.0025
Svi_6.195	0.0003	0.0100
Svi_6.153	0.0052	0.0009
Svi_6.159	0.0349	0.0003
Svi_6.188	0.0220	0.0009
Svi_6.157	0.0734	0.0000
Svi_6.170	0.0112	0.0037
Svi_6.191	0.0068	0.0043
Svi_6.197	0.0002	0.0000
Svi_6.193	0.0127	0.0016
Svi_6.161	0.0005	0.0001
Svi_6.178	0.0016	0.0000
Svi_6.-9	0.0146	0.0121
Svi_6.163	0.0001	0.0000
Svi_6.180	0.0002	0.0000
Svi_6.151	0.0000	0.0027
Svi_6.128	0.0000	0.0000
Svi_6.182	0.0000	0.0000
Svi_6.166	0.0188	0.0001
Svi_6.124	0.0000	0.0006
Svi_6.199	0.0000	0.0001
Svi_6.184	0.0000	0.0000
Svi_6.174	0.0024	0.0009
Svi_6.165	0.0000	0.0014
Svi_6.149	0.0460	0.0033
Svi_6.210	0.0004	0.0001
Svi_6.202	0.0001	0.0003
Svi_6.136	0.0001	0.0002
Svi_6.145	0.0000	0.0000
Svi_6.132	0.0001	0.0000
Svi_6.207	0.0000	0.0001
Svi_6.138	0.0000	0.0000
Svi_6.212	0.0000	0.0000
Svi_6.208	0.0000	0.0004
Svi_6.143	0.0000	0.0001
Mpf_4.215	0.0011	0.0004
Mpf_4.217	0.0102	0.0099
Mpf_4.223	0.0027	0.0165

Mpf_4.191	0.0025	0.0064
Mpf_4.206	0.0000	0.0000
Mpf_4.189	0.0004	0.0018
Mpf_4.225	0.0003	0.0006
Mpf_4.214	0.0003	0.0123
Mpf_4.219	0.0008	0.0068
Mpf_4.212	0.0040	0.0000
Mpf_4.221	0.0018	0.0014
Mpf_4.210	0.0002	0.0008
Mpf_4.193	0.0137	0.0011
Mpf_4.208	0.0000	0.0002
Mpf_4.231	0.0000	0.0002
Mpf_4.-9	0.0003	0.0155
Mpf_4.229	0.0004	0.0002
Mpf_4.185	0.0000	0.0000
Mpf_4.200	0.0000	0.0000
Mpf_4.198	0.0003	0.0000
Mpf_4.183	0.0000	0.0000
Mpf_4.233	0.0003	0.0000
Mpf_4.187	0.0012	0.0051
Mpf_4.227	0.0001	0.0081
Mpf_4.195	0.0001	0.0000
Mpf_4.181	0.0007	0.0001
Mpf_4.235	0.0000	0.0000
Mpf_4.196	0.0000	0.0000
Mpf_4.204	0.0003	0.0009
Mpf_4.237	0.0000	0.0000
Mpf_6.143	0.0041	0.0000
Mpf_6.147	0.0038	0.0003
Mpf_6.138	0.0000	0.0032
Mpf_6.151	0.0005	0.0099
Mpf_6.134	0.0002	0.0156
Mpf_6.-9	0.0000	0.0103
Mpf_6.156	0.0001	0.0002
Mpf_6.160	0.0000	0.0000
Mpf_6.122	0.0003	0.0062
Mpf_6.130	0.0000	0.0000
Mpf_6.118	0.0001	0.0000

Supporting Figures

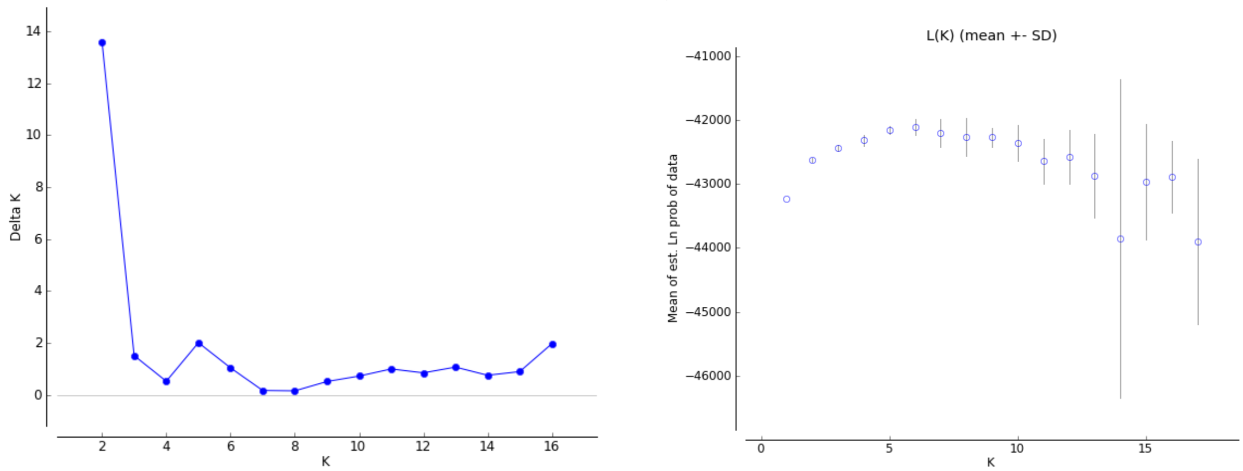

Supplemental Figure S1. Delta K and mean Ln(K) for each value of K (1-17) from STRUCTURE Harvester of STRUCTURE analysis on all yellow perch samples (Fig. 3A).

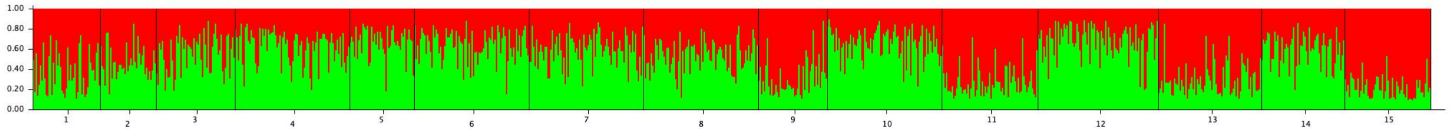

Supplemental Figure S2. STRUCTURE analysis on all yellow perch samples at K=2 using no priors to inform the analysis. Populations are in same order as in Fig 3A.

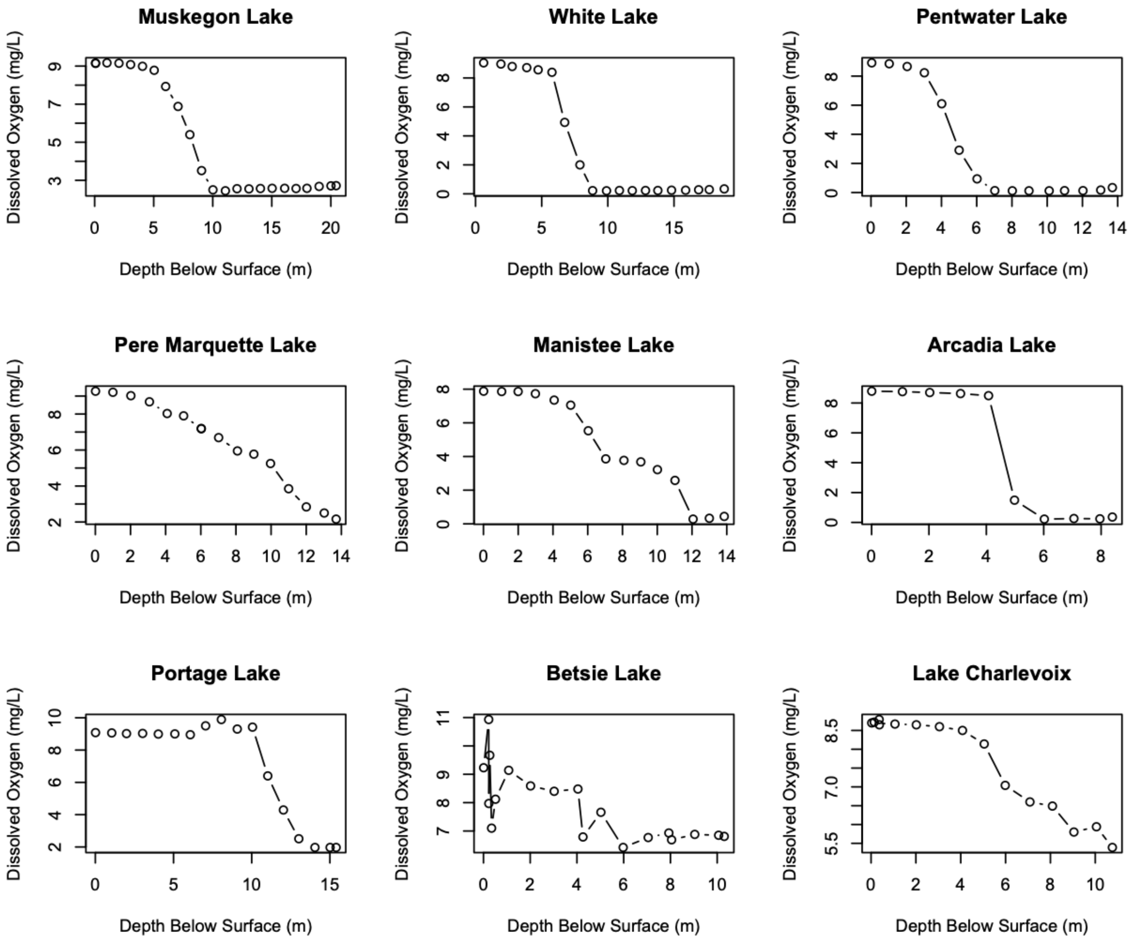

Supplemental Figure S3. Dissolved oxygen as a function of depth below the surface of nine DRM lakes in Summer 2015. Lakes arranged left to right and top to bottom in order of most southern to most northern lake. All lakes measured between 8/5 and 8/18/2015.
